# Effects of Empagliflozin on Sarcopenia Risk, Body Composition, and Muscle Strength in Type 2 Diabetes: A 24-Week Real-World Observational Study

**DOI:** 10.3390/medicina61071152

**Published:** 2025-06-26

**Authors:** Deniz Çetin, Elif Bilgili, Ömer Komaç, Merve Yetişken, Engin Güney

**Affiliations:** Department of Endocrinology and Metabolism, Faculty of Medicine, Aydın Adnan Menderes University, Aydın 09000, Turkey

**Keywords:** empagliflozin, SGLT2 inhibitors, sarcopenia, muscle strength, body composition, type 2 diabetes

## Abstract

*Background and Objectives*: Sodium-glucose cotransporter 2 (SGLT2) inhibitors are increasingly used in type 2 diabetes (T2D) due to their cardiorenal benefits and weight-lowering effects. However, concerns have emerged regarding their potential adverse impact on lean mass and muscle strength particularly in patients at risk for sarcopenia. This study aimed to evaluate the effects of empagliflozin on skeletal muscle mass. Secondary objectives were to assess changes in glycemic control, body weight, fat mass and handgrip strength. *Materials and Methods*: In this 24-week real-world observational study, 31 adult patients with T2D were assigned to either empagliflozin or non-SGLT2i treatment groups. Patients did not receive a high-protein diet, a resistance exercise program or any other weight-reducing medications such as glucagon-like peptide-1 (GLP-1)-based therapies. Anthropometric measurements, body composition via bioelectrical impedance analysis (BIA), and handgrip strength testing were performed at baseline and after 6 months. Sarcopenia was defined according to EWGSOP2 criteria. *Results*: The empagliflozin group showed significant improvements in HbA1c, fasting plasma glucose, body weight, waist circumference, and fat mass (*p* < 0.05 for all). No significant changes were observed in the empagliflozin group after 6 months in appendicular skeletal muscle mass index (from 7.81 ± 1.33 kg/m^2^ to 7.84 ± 1.38 kg/m^2^, *p* = 0.154). No statistically significant changes were observed in handgrip strength in either group. *Conclusions*: Empagliflozin treatment over six months led to favorable changes in metabolic parameters and fat mass without detrimental effects on skeletal muscle mass or muscle strength. In clinical practice, the selection of antidiabetic therapies should consider individual glycemic targets, cardiovascular and renal risks, weight management, comorbidities and sarcopenia risk. Resistance exercises and adequate dietary protein intake should be recommended to preserve muscle mass in at-risk patients. Larger randomized trials are needed to confirm the long-term effects of SGLT2 inhibitors on body composition particularly in older adults.

## 1. Introduction

Diabetes is a chronic disease that continues to pose a global health challenge due to its rapidly increasing prevalence and associated mortality. According to the 2025 Diabetes Atlas of the International Diabetes Federation (IDF), it is estimated that there are 589 million individuals aged 20–79 years living with diabetes worldwide, representing 11.1% of the adult population in this age group. More than 90% of these cases are type 2 diabetes (T2D). IDF data indicate that Turkey has the highest number of people with diabetes among European countries with an estimated 9.6 million individuals affected. The age-standardized prevalence of diabetes in Turkey is 16.5%, ranking first in Europe [1]. The TURDEP-I and TURDEP-II epidemiological studies have demonstrated a 90% increase in diabetes prevalence in Turkey over a 12-year period [2]. What further exacerbates this concerning picture is the low rate of glycemic control among patients receiving treatment [3].

Sarcopenia is a syndrome characterized by the progressive and generalized loss of skeletal muscle mass, quality and strength, leading to an increased risk of falls, fractures, physical disability, and mortality [4]. Although initially associated with aging, sarcopenia is now recognized to begin earlier in life. After the age of 70, the estimated rate of muscle mass loss ranges from 13% to 24% per decade [5]. Risk factors for sarcopenia include cognitive dysfunction, malnutrition, physical inactivity, cancer, major surgical interventions, cardiovascular and respiratory diseases, chronic kidney disease, cirrhosis, hormonal disorders, diabetes and its complications, and obesity [6].

Muscle homeostasis is maintained by a balance between anabolic and catabolic molecular signaling pathways. The anabolic pathway is regulated by the serine/threonine kinase Akt/mTOR cascade, while the catabolic pathway involves FOXO1, NF-κB, the ubiquitin–proteasome system, and myostatin [7]. The most well-known mechanism for the development of sarcopenia in individuals with diabetes is the loss of anabolic effects on skeletal muscle due to insulin deficiency or reduced insulin sensitivity [8]. Furthermore, the accumulation of senescent cells and increased visceral adiposity contribute to chronic low-grade systemic inflammation, which is characterized by elevated levels of proinflammatory cytokines such as IL-1, IL-6, and TNF-alpha. These cytokines enhance the production of reactive oxygen species, leading to the activation of the ubiquitin–proteasome cascade and subsequent muscle proteolysis [9]. The presence of both macrovascular and microvascular complications of diabetes, including retinopathy, nephropathy, and neuropathy, also plays a role in the development of sarcopenia. Epidemiological studies have reported the prevalence of sarcopenia in patients with T2D to range widely—between 7% and 29% across different populations [10].

Combating obesity is one of the most effective strategies for the prevention and management of T2D. In patients with diabetes and obesity, who are at increased risk for sarcopenia, concerns have emerged regarding the potential for weight-reducing antidiabetic agents to exacerbate muscle loss. Conflicting results in clinical studies investigating the relationship between SGLT2 inhibitors and sarcopenia have prompted further research in this area. Therefore, in this study, we aimed to investigate the effects of adding empagliflozin to the treatment regimen of T2D patients who were not receiving SGLT2 inhibitors or glucagon-like peptide-1 (GLP-1) analogs, focusing on its impact on body composition and muscle strength.

## 2. Materials and Methods

### 2.1. Aims and Research Questions

The primary objective of this study was the change in appendicular skeletal muscle mass index after 6 months of empagliflozin treatment. The secondary objectives were to evaluate the effects of empagliflozin on glycemic control (HbA1c, FPG), body weight, waist circumference, fat mass and handgrip strength. The following research questions were addressed:

- Does empagliflozin treatment result in a reduction in skeletal muscle mass or strength?
- What are the changes in fat mass, body weight, and glycemic parameters over a 6-month treatment period with empagliflozin?

### 2.2. Participants

Adults aged 18 to 90 years with T2D, who presented to the Endocrinology Outpatient Clinic of Aydın Adnan Menderes University, Faculty of Medicine between March and May 2024, and who were using oral antidiabetic drugs (OADs) and/or insulin except for SGLT2 inhibitors, GLP-1 receptor agonists (GLP-1 RA) or dual GIP/GLP-1 receptor agonists (GIP/GLP-1 RA), with a body mass index (BMI) > 18.5 kg/m^2^, were included in the study.

Exclusion criteria were as follows: type 1 diabetes (T1D), maturity-onset diabetes of the young (MODY), pregnancy or lactation, estimated glomerular filtration rate (eGFR) < 20 mL/min/1.73 m^2^ or patients undergoing dialysis, chronic alcoholism, cirrhosis, active cancer or follow-up period < 5 years after cancer treatment, history of pancreatic surgery or pancreatitis, medical or surgical treatment for obesity, Cushing’s disease, untreated hypothyroidism or hyperthyroidism, stroke, severe dementia, neuromuscular and rheumatologic diseases, any medical condition requiring prolonged hospitalization, the use of SGLT2 inhibitors, GLP-1 RA, GIP/GLP-1 RA within the past 6 months, and the use of medications known to cause myopathy (e.g., corticosteroids, colchicine, penicillamine).

### 2.3. Study Protocol

The T2D patients included in the study were divided into two non-randomized groups: the empagliflozin group and the non-SGLT2 inhibitor group. Treatments were arranged by the clinician in accordance with the 2024 American Diabetes Association (ADA) Standards of Medical Care in Diabetes, considering glycemic targets and individual risk factors such as cardiovascular disease, nephropathy, obesity, and susceptibility to hypoglycemia as well as drug indications and contraindications [11]. Patients in both groups continued their existing OAD and/or insulin treatments. Patients in the empagliflozin group were initiated on 10 mg/day of empagliflozin, which was maintained for 6 months without any dose adjustment. Patients in the non-SGLT2 inhibitor group were using at least one of the following OADs: metformin, DPP4 inhibitors (linagliptin, vildagliptin, sitagliptin), pioglitazone, gliclazide and acarbose. In addition, patients in the non-SGLT2 inhibitor group were not started on GLP-1 RA, GIP/GLP-1 RA or any other weight-reducing agents during the clinical trial.

Participants received individualized lifestyle counseling delivered by a dietitian with the goal of a 500 kcal/day dietary deficit and were advised to increase their physical activity to at least 150 min/week without a specific strength training protocol.

All patients underwent measurements of height, weight, and waist circumference, handgrip strength testing, and bioelectrical impedance analysis (BIA) as well as laboratory tests including fasting plasma glucose (FPG), HbA1c, and serum creatinine levels both at baseline and at week 24 of the study. All measurements were conducted by the same investigator at both time points.

Handgrip strength, a simple and reliable method for assessing muscle strength, was measured using a validated device: the Camry EH101 digital hand dynamometer (Zhongshan Camry Electronic Co., Ltd., Zhongshan, Guangdong, China) [12]. Body composition analysis, including skeletal muscle mass (SMM), lean mass (LM), and fat mass (FM), was conducted using InBody 230, a direct segmental multi-frequency bioelectrical impedance analyzer (InBody Co., Ltd., Seoul, Republic of Korea). This analyzer performs 10 impedance measurements using two frequencies (20 kHz and 100 kHz) across five body segments (right arm, left arm, trunk, right leg, and left leg). According to the EWGSOP2 criteria, an appendicular skeletal muscle mass index (ASM/height^2^) of <5.5 kg/m^2^ for women and <7 kg/m^2^ for men was used as the cut-off for sarcopenia. Handgrip strength values below 16 kg for women and 27 kg for men were considered low [13].

The study protocol was approved by the Clinical Research Ethics Committee of Aydın Adnan Menderes University Faculty of Medicine on 13 February 2024 with the decision number 493812 (Protocol No: 2024/29). The study was conducted in accordance with the Declaration of Helsinki, and informed consent was obtained from all participants.

The manuscript was prepared in accordance with the STROBE (Strengthening the Reporting of Observational Studies in Epidemiology) reporting guideline. The completed STROBE checklist is provided as Supplementary Material S1.

### 2.4. Statistical Analysis

Statistical analyses were performed using SPSS version 22.0 (Statistical Package for the Social Sciences; SPSS Inc., Chicago, IL, USA). Categorical variables were expressed as numbers and percentages (*n*, %), while continuous variables were presented as mean ± standard deviation (SD). The conformity of the data to the normal distribution was checked with the Shapiro–Wilk test. Baseline characteristics between groups were compared using Pearson’s chi-squared test for categorical variables and the Mann–Whitney U test for continuous variables. The Wilcoxon signed-rank test was used to evaluate treatment-related changes within groups. A *p*-value of <0.05 was considered statistically significant.

## 3. Results

Among the patients who were initiated on empagliflozin, 52% (*n* = 11) were female and 48% (*n* = 10) were male, whereas in the non-SGLT2 inhibitor group, 70% (*n* = 7) were female and 30% (*n* = 3) were male. The mean age of the empagliflozin group was 54.6 ± 11.7 years, while that of the non-SGLT2i group was 61.1 ± 11 years. No statistically significant differences were observed between the groups in terms of age or sex. However, the duration of diabetes was significantly longer in the empagliflozin group (9.8 vs. 3.7 years, *p* = 0.013). Chronic complications of diabetes were present in 33.3% (*n* = 7) of patients in the empagliflozin group, while none were reported in the non-SGLT2i group. No significant differences were found between the groups in serum creatinine or estimated glomerular filtration rate (eGFR). In the empagliflozin group, 90% (*n* = 19) of patients were using at least one OAD, and 43% (*n* = 9) were on insulin therapy. In contrast, all patients in the non-SGLT2i group (*n* = 10) were on at least one OAD, and none were receiving insulin. The mean HbA1c level in the empagliflozin group was 8.6% compared to 6.8% in the non-SGLT2i group, which is a statistically significant difference (*p* = 0.042). The baseline characteristics of the patients are presented in Table 1.

Anthropometric analysis revealed that the mean BMI in the empagliflozin group (31.5 ± 4 kg/m^2^) was higher than that of the non-SGLT2i group (28.8 ± 5 kg/m^2^), though this difference was not statistically significant (*p* = 0.190). Among female patients, waist circumference was significantly higher in the empagliflozin group compared to the non-SGLT2i group (*p* = 0.041), whereas no significant difference was observed in male patients. No statistically significant differences were observed between the groups in terms of handgrip strength, appendicular skeletal muscle mass (ASM), or appendicular skeletal muscle mass index (ASMI). Overall, 12.9% (*n* = 4) of the 31 patients were identified as having sarcopenia based on the defined criteria. The number of sarcopenic patients did not differ significantly between the groups (*p* > 0.05) (Table 1).

Changes in HbA1c, fasting plasma glucose (FPG), handgrip strength, and body composition parameters assessed by BIA over the 6-month follow-up period were compared between the empagliflozin and non-SGLT2i groups (Table 2).

In the empagliflozin group, significant improvements were observed in both FPG and HbA1c levels at 6 months (*p* = 0.014 and *p* = 0.049, respectively), while no significant changes were noted in the non-SGLT2i group. Both groups exhibited significant reductions in body weight after 6 months (empagliflozin group: from 81.4 ± 15.3 kg to 78.8 ± 14.1 kg, *p* = 0.016; non-SGLT2i group: from 75.7 ± 10 kg to 73.4 ± 10.2 kg, *p* = 0.008). Waist circumference significantly decreased in the empagliflozin group (from 109.5 ± 9.8 cm to 102.8 ± 10 cm, *p* = 0.025), whereas no significant change was detected in the non-SGLT2i group (from 99.6 ± 12.9 cm to 98.6 ± 7.6 cm, *p* = 0.593).

Fat mass significantly decreased in the empagliflozin group after 6 months (from 30.9 ± 8.6 kg to 28.8 ± 8.1 kg, *p* = 0.04). Although a reduction in fat mass was also observed in the non-SGLT2i group (from 30.4 ± 11.1 kg to 28.0 ± 8.3 kg), this change did not reach statistical significance (*p* = 0.059).

Regarding sarcopenia parameters, no significant changes were observed in the empagliflozin group after 6 months in ASM (from 20.73 ± 5.5 kg to 20.78 ± 5.5 kg, *p* = 0.164) or ASMI (from 7.81 ± 1.33 kg/m^2^ to 7.84 ± 1.38 kg/m^2^, *p* = 0.154). Similarly, no statistically significant changes were observed in SMM, ASM, or ASMI in the non-SGLT2i group after 6 months. Although not statistically significant, both groups showed an increase in muscle strength (handgrip) at 6 months compared to baseline.

## 4. Discussion

Cardiovascular (CV) outcome trials have shown that sodium–glucose cotransporter 2 (SGLT2) inhibitors provide both cardiac and renal protection, leading to significant changes in T2D treatment guidelines. The American Diabetes Association (ADA) recommends the use of SGLT2 inhibitors in patients with established atherosclerotic cardiovascular disease, high CV risk, heart failure, or chronic kidney disease regardless of HbA1c levels [11]. SGLT2 inhibitors promote glucosuria by inhibiting glucose reabsorption in the proximal renal tubule, and the associated suppression of insulin secretion and stimulation of glucagon secretion protect against hypoglycemia [14]. Their weight-reducing effect also offers an advantage in the treatment of obese T2D patients. Based on these principles, the patient groups in our study were structured accordingly. Consequently, the empagliflozin group included patients with poorer glycemic control, longer diabetes duration, insulin use, and coexisting chronic complications such as cardiovascular diseases.

In this study, a 6-month treatment with empagliflozin 10 mg/day led to an average HbA1c reduction of 1.2%. In addition to improved glycemic control, significant reductions in body weight and waist circumference were observed. The favorable metabolic outcomes observed in the empagliflozin group after 6 months may be attributed not only to the pharmacologic effect but also to patient adherence to lifestyle recommendations such as diet and physical activity.

Bioelectrical impedance analysis (BIA), a non-invasive, low-cost, and practical method, was used in our study for the quantitative assessment of muscle mass [15]. Since muscle mass correlates with body size, adjusted parameters such as ASM/height^2^ were evaluated in addition to SMM and ASM [13,16]. No statistically significant changes were found in LM, SMM, ASM, or ASMI after 6 months of empagliflozin treatment.

The hypothesis that SGLT2 inhibitors may induce sarcopenia stems from the idea that the glucosuria-induced gluconeogenesis not only triggers lipolysis but may also initiate proteolysis in skeletal muscle by utilizing amino acids as substrates [17]. Previous studies have yielded mixed results: a multicenter, randomized, open-label study comparing ipragliflozin and sitagliptin reported significant loss in both body fat percentage and skeletal muscle mass index after 12 weeks of ipragliflozin treatment [18]. In contrast, Sugiyama et al. reported that dapagliflozin treatment did not reduce skeletal muscle mass index or psoas major area in a study using both BIA and CT. Moreover, a significant increase in L3 paravertebral muscle attenuation on CT was observed in the dapagliflozin group, suggesting a reduction in intramuscular fat accumulation [19]. Another study comparing the addition of empagliflozin or linagliptin to premixed insulin therapy in T2D patients found that while empagliflozin was associated with a reduction in muscle mass, neither drug caused significant changes in skeletal muscle mass [20].

A recent meta-analysis reported that SGLT2 inhibitor use in diabetic patients was associated with significant reductions in LM, SMM, and SMI, raising concerns about increased sarcopenia risk accompanying weight loss [21]. However, moderate heterogeneity (65%) was noted among the 12 studies evaluating LM, and only four studies assessed SMI using BIA (*n* = 137 SGLT2i users, 137 non-users).

In our study, a significant reduction in fat mass was observed in the empagliflozin group at the end of the 6-month treatment. SGLT2 inhibitors are known to promote weight loss primarily by reducing fat mass [22]. This effect is attributed not only to glucosuria and increased energy expenditure but also to the enhanced β-oxidation of fatty acids [23]. It is believed that SGLT2 inhibitors may also improve adipocyte dysfunction in visceral fat by decreasing leptin, visfatin, and plasminogen activator inhibitor-1 while increasing adiponectin levels, thereby contributing to reduced visceral adiposity [24,25].

Muscle strength is the most reliable indicator of muscle function. Low muscle strength is the first criterion in diagnosing sarcopenia, and such patients are classified as having probable sarcopenia. The diagnosis is confirmed when low muscle quantity or quality is also demonstrated [13]. Handgrip strength is considered a better predictor of adverse outcomes than muscle mass [26]. For this reason, all patients in our study underwent handgrip strength testing. In the empagliflozin group, handgrip strength increased after 6 months compared to baseline. Although the change was not statistically significant, the improvement may reflect enhanced muscle function due to reduced intramuscular fat infiltration. When evaluating the two female patients in the empagliflozin group who were diagnosed with sarcopenia at baseline (aged 69 and 78), a slight decrease in ASMI was observed, but both showed an increase in handgrip strength (case 1: 14.8 kg to 17.2 kg; case 2: 10.8 kg to 14.7 kg). Similarly, in the study by Sano et al., after at least 4 weeks of SGLT2 inhibitor therapy, both male and female patients with T2D showed significant improvements in handgrip strength [27].

### 4.1. Perspective for Clinical Practice

In addition to their well-established cardiorenal benefits, glucagon-like peptide-1 receptor agonists (GLP-1 RAs), which are increasingly preferred as effective treatments for obesity, have not demonstrated consistent evidence of disproportionate lean mass loss or reductions in muscle strength in body composition analyses [28]. In a sub-analysis of the SUSTAIN-8 trial evaluating the efficacy and safety of subcutaneous semaglutide, changes in body composition were assessed via dual-energy X-ray absorptiometry (DEXA) over 52 weeks in a comparison of canagliflozin 300 mg/day and semaglutide 1.0 mg/week. Both treatment arms showed reductions in total fat and lean mass, yet by week 52, the proportion of total lean mass increased in both groups [29]. In a real-world study of subcutaneous semaglutide, although some loss of lean mass was observed alongside weight reduction over 6 and 12 months of follow-up, clinically meaningful improvements were noted in glycemic control, blood pressure, lipid profiles, and quality of life parameters [30]. In the SURMOUNT-1 trial, which investigated the effects of the dual GIP/GLP-1 receptor agonist tirzepatide on body composition in individuals with obesity or overweight, it was found that 75% of weight loss was attributable to fat mass and 25% to lean mass with no significant difference compared to placebo [31].

In clinical practice, when prescribing weight-lowering antidiabetic agents, strategies to mitigate the risk of sarcopenia should include resistance exercises and increased daily dietary protein intake [32]. The recommended exercise types for preserving and enhancing muscle strength are resistance exercises performed alone or in combination with aerobic or balance exercises [33]. A minimum protein intake of 1–1.2 g/kg/day through diet is advised [34]. Naturally, the treatment process should be managed in collaboration with a dietitian, considering patients’ comorbidities.

### 4.2. Limitations

This study has several limitations, including a small sample size, lack of randomization, and open-label design. The stringent exclusion criteria used to eliminate potential confounders of sarcopenia, along with the widespread use of GLP-1 analogs and/or SGLT2 inhibitors in routine care at our tertiary center, may have contributed to the limited number of eligible patients. The relatively small sample size limits the robustness and generalizability of the results of this study. Furthermore, the empagliflozin group included patients with more advanced disease, complications, and a greater use of OADs and insulin, which may introduce recruitment bias. Considering that the study was designed as a real-life study and SGLT2 inhibitors are recommended with a strong level of evidence for the treatment of T2D with cardiorenal risk factors, recruitment bias could not be avoided. Although the mean age was similar between groups, the elderly population was not sufficiently represented. Since the elderly population is already at risk for sarcopenia, clinicians should be careful when using SGLT2 inhibitors in the elderly. Unfortunately, we do not have data on patient adherence to lifestyle modifications. It should be taken into consideration that this may have affected the outcomes of the study.

## 5. Conclusions

When selecting medical treatment for patients with T2D, individual glycemic targets, cardiovascular and renal risk factors, weight management, comorbidities, and side effect profiles should all be carefully considered. The potential reduction in lean mass accompanying fat mass loss with SGLT2 inhibitors or GLP-1-based therapies warrants caution in patients at risk of sarcopenia. In such individuals, dietary interventions and resistance exercise programs aimed at preserving muscle mass should be implemented. In this study, 6 months of empagliflozin treatment resulted in significant reductions in body weight, waist circumference, and fat mass without any adverse effects on muscle mass or muscle strength in patients with T2D. Larger, randomized studies are needed to evaluate the long-term effects of SGLT2 inhibitors on body composition particularly in older T2D patients at risk for sarcopenia.

## Figures and Tables

**Table 1:** Baseline characteristics of the patient groups.

	Empagliflozin	Non-SGLT2 İnhibitor	*p* Value
Age	54.6 ± 11.7	61.1 ± 11	0.176 *
Gender (female/male)	11/10	7/3	0.353 **
Duration of diabetes, years	9.8 ± 8.6	3.7 ± 5	0.013 *
Chronic complications, *n* (%)	7 (33.3%)	0	0.038 **
Use of other OADs, *n* (%)	19 (90.4%)	10 (100%)	0.313 **
Use of insulin, *n* (%)	9 (42.8%)	0	0.014 **
HbA1c, %	8.8 ± 2.6	6.8 ± 1.1	0.042 *
Fasting plasma glucose, mg/dL	216 ± 126	122 ± 42	0.003 *
Serum creatinine, mg/dL	0.8 ± 0.2	0.7 ± 0.1	0.157 *
Glomerular filtration rate (eGFR), mL/dk/1.73 m^2^	92 ± 21	94 ± 13	0.916 *
Body mass index, kg/m^2^	31.5 ± 4	28.8 ± 5	0.190 *
Waist circumference, cm			
Female	111 ± 10.8	97.7 ± 12	0.041 *
Male	107.9 ± 8.9	104 ± 16.5	1.000 *
Handgrip strength, kg			
Female	23.5 ± 6.9	24.2 ± 5.7	0.964 *
Male	37.7 ± 7.1	39.1 ± 7.2	0.866 *
Appendicular skeletal muscle mass (ASM), kg			
Female	18.14 ± 3.32	15.08 ± 4.14	0.113 *
Male	25.31 ± 2.39	23.73 ± 2.8	0.612 *
Appendicular skeletal muscle mass index (ASMI), kg/m^2^			
Female	7.36 ± 0.89	6.44 ± 1.28	0.077 *
Male	8.69 ± 0.71	8.58 ± 0.94	0.735 *
Sarcopenia, *n* (%)	2 (9.5%)	2 (20%)	0.335 **

*: The Mann–Whitney U test was performed. **: Pearson’s chi-squared test was performed.

**Table 2:** Changes in body composition and metabolic parameters at 6 months of treatment.

	Empagliflozin			Non-SGLT2 Inhibitor		
	Baseline	6 Months	* *p* Value	Baseline	6 Months	* *p* Value
Body weight (kg)	81.4 ± 15.3	78.8 ± 14.1	0.016	75.7 ± 10	73.4 ± 10.2	0.008
Body mass index (kg/m^2^)	31.5 ± 4	30.1 ± 4.2	0.33	28.8 ± 5	28.6 ± 3.3	0.878
Lean mass (kg)	50.5 ± 11.2	50 ± 11.3	0.159	45.3 ± 7.2	45.3 ± 11.8	0.445
Skeletal muscle mass (SMM) (kg)	27.98 ± 6.85	27.70 ± 6.89	0.225	24.85 ± 4.34	24.66 ± 7.82	0.507
Appendicular skeletal muscle mass (ASM) (kg)	20.73 ± 5.5	20.78 ± 5.5	0.164	19.41 ± 4.6	19.09 ± 4.5	0.284
Appendicular skeletal muscle mass index (ASMI) (kg/m^2^)	7.81 ± 1.33	7.84 ± 1.38	0.154	7.48 ± 1.18	7.35 ± 1.2	0.284
Fat mass (kg)	30.9 ± 8.6	28.8 ± 8.1	0.04	30.4 ± 11.1	28 ± 8.3	0.059
Total body water (kg)	37.1 ± 8.2	36.8 ± 8.3	0.218	33.4 ± 5.2	33.4 ± 8.7	0.386
Waist circumference (cm)	109.5 ± 9.8	102.8 ± 10	0.025	99.6 ± 12.9	98.6 ± 7.6	0.593
Handgrip strength (kg)	30.3 ± 9.9	32.2 ± 9.2	0.602	28.7 ± 9.2	30.5 ± 11.1	0.878
HbA1c (%)	8.8 ± 2.6	7.6 ± 1.4	0.049	6.8 ± 1.1	6.8 ± 0.8	0.858
Fasting plasma glucose (mg/dL)	216 ± 126	126 ± 30	0.014	122 ± 42	109 ± 20	0.610

*: The Wilcoxon signed-rank test was performed.

## Data Availability

The data presented in this study are available on request from the corresponding author.

## Supplementary Materials

The following supporting information can be downloaded at https://www.mdpi.com/article/10.3390/medicina61071152/s1.

## Abbreviations

The following abbreviations are used in this manuscript:

ASMI	appendicular skeletal muscle mass index
BIA	bioelectrical impedance analysis
BMI	body mass index
EWGSOP	European Working Group on Sarcopenia in Older People
FOXO1	forkhead box protein O1
FPG	fasting plasma glucose
GIP	glucose-dependent insulinotropic polypeptide
GLP-1	glucagon-like peptide-1
IL	interleukin
NF-κB	nuclear factor-κB
OADs	oral antidiabetic drugs
SGLT2i	sodium-glucose cotransporter 2 inhibitor
T2D	type 2 diabetes
TNF	tumor necrosis factor
